# The roles of fractalkine signaling in neurodegenerative disease

**DOI:** 10.1186/1750-1326-7-S1-L21

**Published:** 2012-02-07

**Authors:** Bruce Lamb

**Affiliations:** 1 Staff Scientist, Department of Neurosciences, The Lerner Research Institute, The Cleveland Clinic Foundation, Cleveland, Ohio, USA

## Background

The primary neuropathological characteristics of AD include intracellular, filamentous inclusions of hyperphosphorylated microtubule associated protein tau (MAPT) in neurons (neurofibrillary tangles, NFTs) and extracellular deposits of the beta-amyloid peptide (A-Beta) in senile plaques. While A-Beta plaques are unique to AD, NFTs are observed in a wide variety of neurodegenerative diseases, including AD, frontotemporal dementia, cortical basal degeneration and other disorders, collectively termed tauopathies. Microglia, the primary immune effector cells in the brain, continually monitor the tissue parenchyma for pathological alterations and become activated in AD and other tauopathies. Increasing evidence suggests that microglia contribute to the pathopysiology of AD including; correlational studies in human AD and mouse models, retrospective epidemiological studies demonstrating that use of nonsteroidal anti-inflammatory drugs (NSAIDs) substantially reduces AD risk, genome-wide association studies of human AD and studies utilizing genetically modified mouse models. Neuronal-microglial communication through the chemokine fractalkine (CX3CL1) and its cognate receptor, CX3CR1, plays a critical role in neuroinflammation and neuroprotection. First, CX3CL1 is highly expressed by neurons while CX3CR1 is only expressed by microglia within the CNS. Second, CX3CL1 is neuroprotective in models of neuroinflammation. Finally, studies of *Cx3cr1* knockout mice revealed enhanced neurodegeneration in mouse models of amyotrophic lateral sclerosis (ALS) and Parkinson’s Disease (PD).

## Methods

To examine the role of CX3CL1-CX3CR1 signaling in Alzheimer’s disease, we examined the effects of CX3CR1 deficiency on both A-Beta and MAPT pathologies in the APPPS1 (and R1.40) and hTau mouse models of AD, respectively. Mice were aged and examined for alterations in gene expression, biochemistry, neuropathology and behavior.

## Results

Surprisingly, CX3CR1 deficiency resulted in a gene dose-dependent reduction in A-Beta deposition in both the APPPS1 and R1.40 transgenic mouse models of A-Beta deposition that was associated with altered microglial activation, reduced numbers of plaque associated microglia and yet enhanced removal of A-Beta. Analysis of CX3CR1 deficient microglia revealed an enhanced capacity for phagocytosis of A-Beta and also increased expression of matrix metalloproteinase 9 (MMP9), an A-beta degrading protease. By contrast, CX3CR1 deficiency in the hTau mouse model of MAPT pathology resulted in enhanced microglial activation and phagocytosis, phosphorylation and aggregation of MAPT and behavioral impairments. Additional *in vitro* experiments demonstrate that microglial activation and CX3CR1 deficiency elevates the level of active p38 MAPK and enhances MAPT hyperphosphorylation within neurons that can be blocked by administration of a specific p38 MAPK inhibitor. In vivo adoptive transfer experiments in which microglia were purified from CX3CR1 deficient hTau mice and transferred to non-transgenic controls, revealed enhanced MAPT phosphorylation demonstrating the cell autonomous nature of the signal.

## Conclusion

Our results suggest that CX3CR1 deficiency has opposing effects on the development of the two primary pathologies observed in AD and suggests that “anti-inflammatory” therapies may have opposite effects on AD pathologies at different stages of disease progression.

